# Anal Canal Duplication in an Adult Female—Case Report and Pathology Guiding

**DOI:** 10.3390/medicina57111205

**Published:** 2021-11-04

**Authors:** Tudor Mateescu, Cristi Tarta, Paul Stanciu, Alis Dema, Fulger Lazar

**Affiliations:** 1 Surgery 2, Department X, Researching Future Chirurgie 2, “Victor Babeș” University of Medicine and Pharmacy, Eftimie Murgu Sq. No. 2, 300041 Timișoara, Romania; Tudor_mat@yahoo.ro (T.M.); lazarfulger@yahoo.com (F.L.); 2 Watford Cancer Centre, Gynecological Oncology Department, West Hertfordshire NHS Hospitals Trust, Watford WD18 0GU, UK; 3 ANAPATMOL Research Center, Pathology, Department II, ” Victor Babeș” University of Medicine and Pharmacy, Eftimie Murgu Sq. No. 2, 300041 Timișoara, Romania; dema_alis@yahoo.com

**Keywords:** anal canal duplication, adult anal duplication, ano-rectal malformations

## Abstract

Anal canal duplication (ACD) is a very rare condition, diagnosed and treated mostly in childhood. Less than 90 cases have been reported in the literature so far. We are presenting a case of a young woman who underwent surgical excision of the duplication when she was 27 years old. The patient was unaware of her condition and was referred from a gynaecological office to the surgical department with a history of perianal discomfort and mucus discharge. Local examination showed an external orifice posterior to the anal opening, on the median line, which had the macroscopic appearance of a secondary anal orifice. The opening was about 0.5 cm in diameter. Exploration of the tract revealed a length of about 4 cm. MRI described the aforementioned tract, parallel to the anal canal, with no other anomalies mentioned. Under spinal anesthesia, with the patient in jackknife position, the accessory anal canal was surgically excised. The pathology report showed the presence of smooth muscle fibers and typical anal glands in the specimen. After a five-year follow-up, the patient showed no recurrence or any other related local symptoms. Absence of perianal abscess from the patient history, along with the macroscopic aspect of the opening similar to a secondary anal orifice on the midline, should raise the suspicion of ACD. Due to the lack of bothersome symptomatology, the patient did not seek any special investigations for her condition until she was in her late twenties. ACD is a very rare condition in adults that might pass unnoticed, but a midline opening posterior to the anus should always raise the suspicion of a secondary anal canal. Surgery is the only cure for this condition with good results after a proper pre-operative workout to reveal others simultaneous malformations.

## 1. Introduction

Anal canal duplications (ACD) are the rarest malformations of the digestive tract, seen and treated mostly in childhood. They are described as a consequence of duplication of the dorsal cloaca in an early developmental stage or a consequence of recanalization of a cloacal membrane excess in late embryonic life [1,2]. Hoda et al. proposed the definition: “the term ACD should be restricted to a single duplication of the anal canal, not including cases with others duplication of the hindgut with or without genito-urinary involvement but including some cases with sacral dysgenesis or congenital ano-rectal malformations” [2]. Around 90 cases have been reported in the literature so far [3]. Sometimes, due to the lack of symptoms or the occurrence of symptoms that may mimic other anorectal conditions, the diagnosis might be overlooked. ACD is 9:1 more common in women than in men and associates other malformations up 36% of the cases [3,4]. Half of the patients are asymptomatic when diagnosed, one third are mildly symptomatic, and one in fifth present with complications [5]. Thus, we present a case of a young woman diagnosed with ACD in her late twenties. She did not have any bothersome symptoms before this age, except for the presence of a second anal orifice and minor mucus discharge.

## 2. Case Report

**Figure 1:**
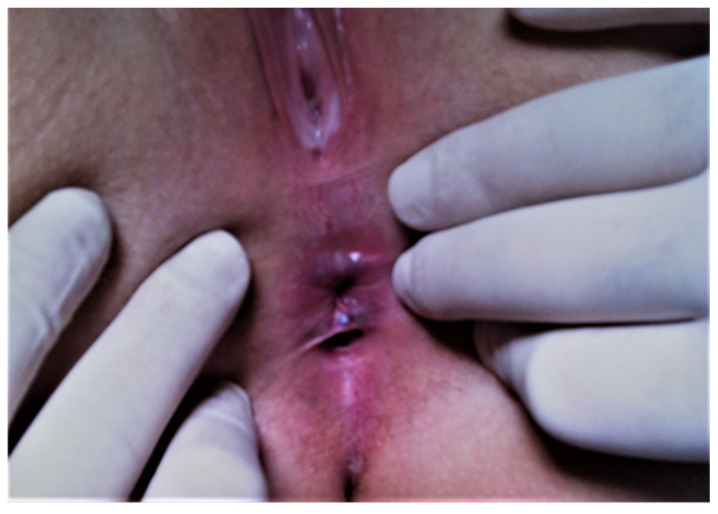
Additional midline opening on the midline posterior to the normal anus.

A 27-year-old woman was seen in the outpatient clinic of the surgical department, with a second anal opening situated along the midline, posterior to the normal anal canal opening (Figure 1). The patient was referred from a gynecological office. She described some episodes of mild discomfort in the area, followed by the secretion of a mucinous-like fluid, with the disappearance of the discomfort after fluid discharge starting two years ago. The appearance of the fluid was whitish and clear. She did not have any significant past medical history and denied any past episodes of abscess.

During the examination, an additional opening was revealed posterior to the anal canal opening, situated on the midline. There was no pain on palpation of the perianal region or any other findings on digital rectal examination of the normal anal canal. The ACD orifice had a smaller diameter than the evaluator’s finger; therefore, only instrumental exploration was performed in order to determine the length of the tract—around 4 cm, and the presence of any intraluminal secretions—none were discovered. Anoscopy showed a normal anal canal with first degree hemorrhoids, and no communication between the two tracts was found when hydrogen peroxide and methylene blue were injected in the ACD.

Patient consent was obtained in order to take pictures of the lesion—the strange, perineal second orifice, which looked like a mini-anus, with a structure mimicking the dentate line—and to have it evaluated by our clinic’s panel of experts. 

After our department meeting, the suspicion of an ACD was raised and an MRI was performed for further evaluation. MRI described a tract with a length of 4 cm situated posterior to the anal canal, with no communication between the two structures.

Possible differential diagnoses were perianal fistula—usually developing on the sides and being accompanied by abscess, or other malformations of the anorectal region, such as dermoid cyst, presacral teratoma, lumbosacral meningocele, spina bifida, and others. MRI ruled out these differential diagnoses. 

The patient was admitted for surgical intervention under regional anesthesia. She was placed in a jackknife position with the legs abducted at 60 degrees. The ACD was removed using electrocautery. Special care was taken to prevent any anal sphincter damage. At the proximal end of the ACD, some small cysts were discovered. The whole tract was excised, and the skin was close after the procedure with absorbable sutures. Postoperative recovery was uneventful, and the patient was discharged on the third post-operative day. 

**Figure 2:**
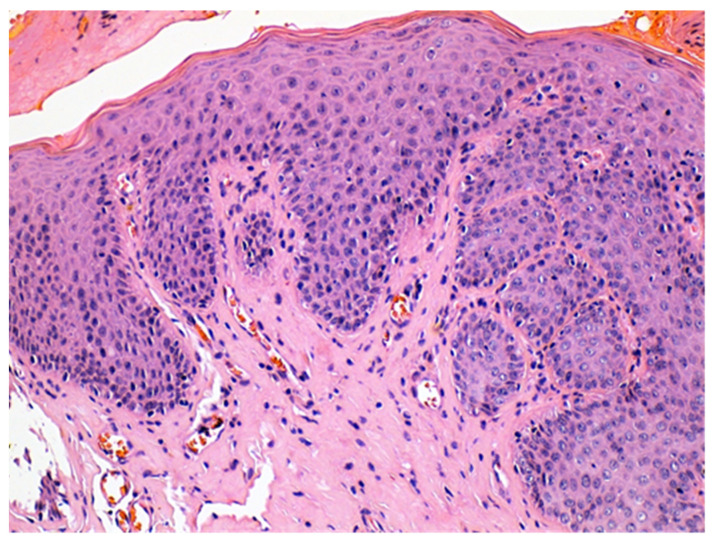
Hyperplastic stratified squamous epithelium. HE × 200.

**Figure 3:**
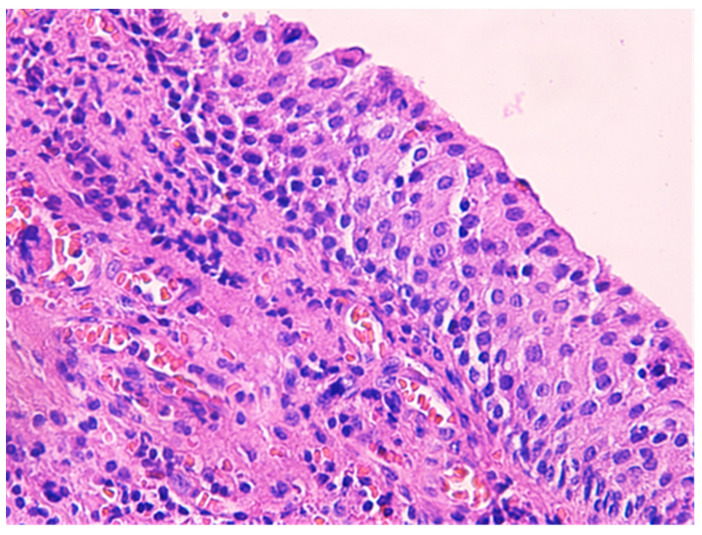
Transition zone-type epithelium. HE × 400.

**Figure 4:**
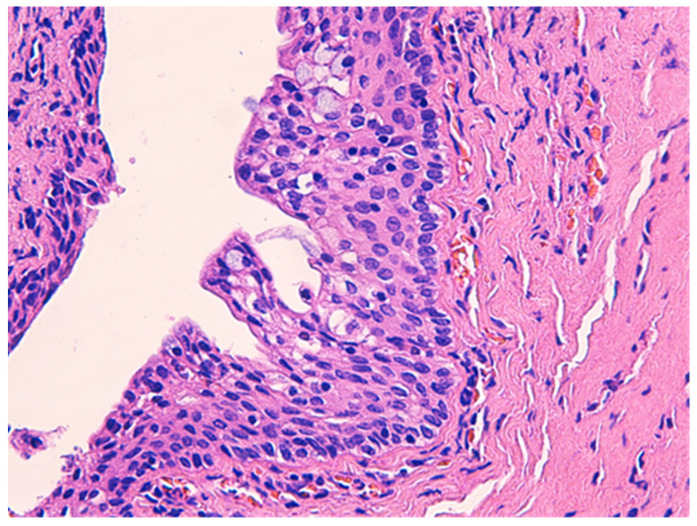
Transition zone-type epithelium with goblet cells within. HE × 400.

**Figure 5:**
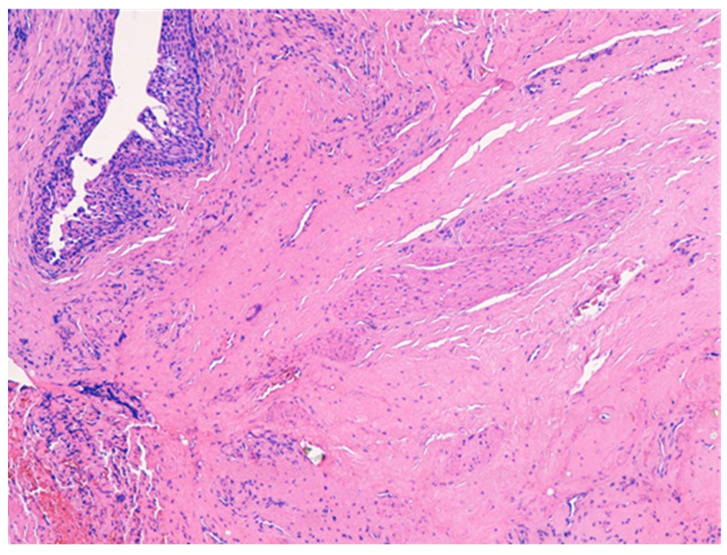
Bundles of smooth muscle cells. HE × 100.

**Figure 6:**
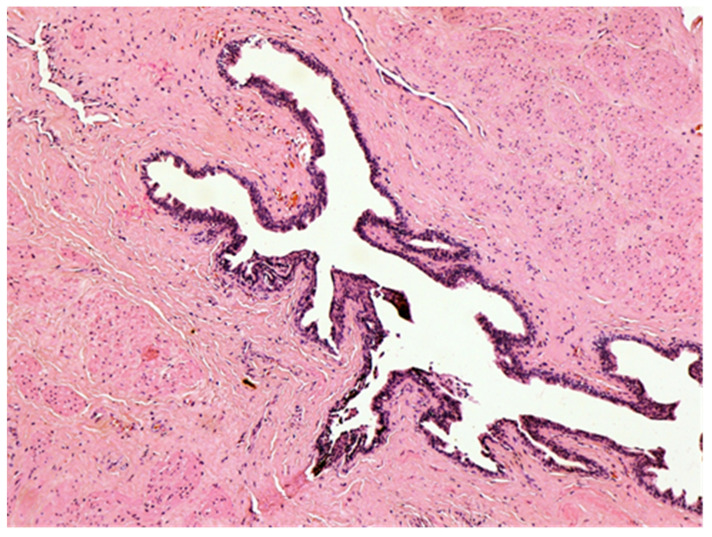
Anal duct. HE × 100.

**Figure 7:**
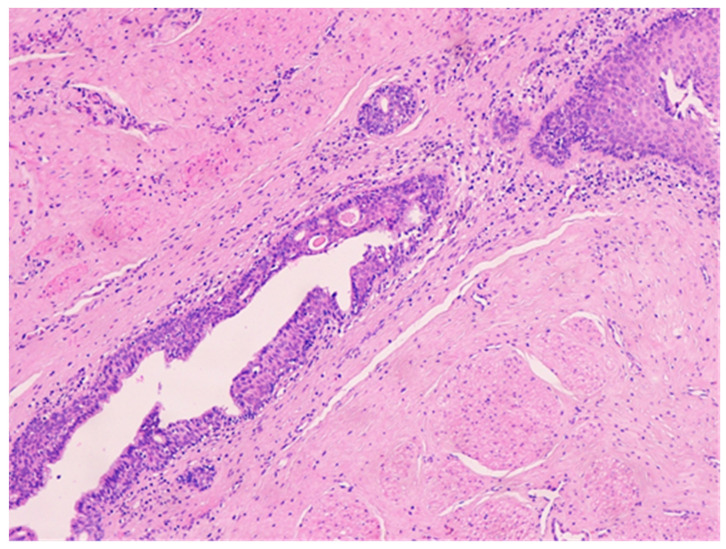
Anal duct with stratified epithelium (transition zone-type) containing microcysts. HE × 100.

**Figure 8:**
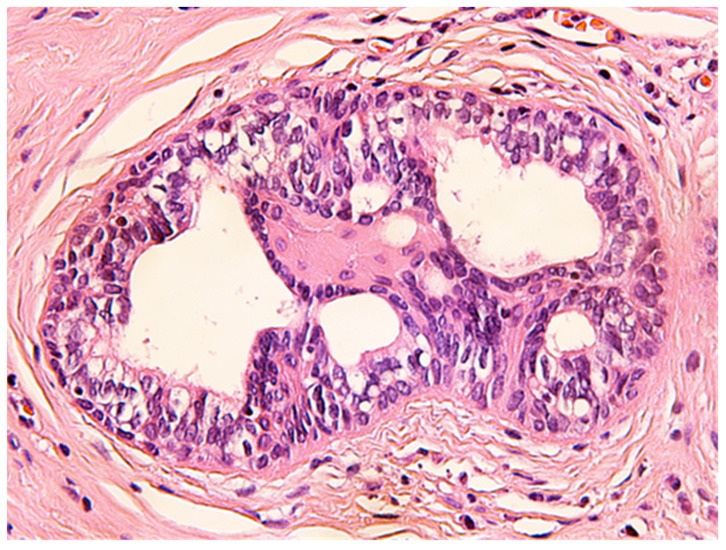
Anal gland with focal squamous metaplasia. HE × 400.

The histologic examination of the specimen revealed the presence of stratified squamous epithelium (Figure 2) and transition zone epithelium (Figure 3). The latter contained isolated or clustered goblet cells (Figure 4), overlying fragments composed of connective, and adipose tissue (including bundles of smooth muscle cells (Figure 5), anal ducts (Figure 6), focally with microcysts formation within the transition zone-type epithelium, and anal glands (Figure 7) with small foci of squamous metaplasia (Figure 8)).

The outcome was favorable, and the patient was completely healed with no recurrence after a five-year follow-up.

## 3. Discussion

ACD is a very rare malformation, in adults it is even rarer because most of the lesions are operated in childhood and infancy. Being so rare, clinicians have difficulties in recognizing ACD, usually mistakenly diagnosing it as a perianal fistula or perianal abscess. Ochiai et al. defined ACD, based upon three features: the presence of squamous epithelium in the caudal end, transitional epithelium in the cranial end, and smooth-muscle cells in the wall of the lesion [6]. The particularity of our case was the presence of anal glands which explain the secretion of fluid. To the best of our knowledge, the presence of anal glands has been reported only in few cases in the literature before.

These complementary features should be evaluated in the context of the anatomical presence of an additional anus-like opening, posterior to the normal anus, usually situated on the midline. The only case in the literature where an ACD opening was described outside of midline at five o’clock was presented by Rezvan et al. [7]. Some authors performed anoscopy on the ACD, [8] but in our case, the narrow opening of the lesion did not allow such an investigation to be performed.

Usually, the parents or the doctors discover the presence of an ACD during early stages of life, but sometimes because of the lack of symptoms, the lesion is noticed only in adulthood, when complications occur. 

Apart from the presence of an additional opening, ACD often becomes symptomatic, with local infection and discharge as the most common signs [4]. Imaging plays a vital role in excluding other pathologies. For example, MRI excludes presacral congenital mal-formations and the documentation of fistulous path between the accessory anal canal and the anus or rectum [9]. One of the conditions associated with ACD were tailgut cysts, in 11% of the cases reported [4]. Tailgut cysts arise from the remnants of the hindgut and determine mainly retrorectal tumors which can lead, among others, to fistula tract formation. Women are more affected than men, but the age of these patients was usually older than those with ACD at diagnostics [9,10]. 

Fistulography can also be used to exclude the presence of fistulous tracts and/or communication with the anal or rectal lumens. Anoscopy can be used in the fashion described in the work-up of our case, with injection of hydrogen peroxide and methylene blue in order to rule out a communication with the anal canal. Echoendoscopy can be a useful tool, allowing the identification of the anal sphincter, the ACD tract, and its dimensions [11]. 

Surgery is the only curative option. Discussions about the therapeutic indications for asymptomatic ACD have been ongoing and are based on a report from Duke and Galvin, [12] released in 1956, regarding the risk of malignant transformation of ACD. No other cases of malignant transformation have been reported since. Other therapeutic indications are bothersome symptomatology and aesthetic considerations. There are two therapeutic options—stripping the mucosa and surgical abolishment of the remaining canal or complete excision of the ACD. We performed a complete excision of the ACD. If there are other associated malformations with the ACD, the surgical cure must provide additional solutions to these concomitant lesions. The mucosa stripping was preferred in ACD with a shorter tract less than 30 mm [13] without other concomitant lesions, and the perineal approach with complete removal was performed by others for the same situation. The additional malformations prompted for more complex surgery, such as a combined perineal and sacral approach, while an anorectal malformation prompted for a posterior sagittal anorectoplasty [14].

## 4. Conclusions

ACD is a very rare condition in adults that might pass unnoticed, but a midline opening posterior to the anus should always raise the suspicion of a secondary anal canal. Surgery is the only cure for this condition with good results after a proper preoperative workout to reveal other simultaneous malformations. Definitive diagnostic has to be supported by the pathology report, confirming the presence of squamous and transition zone-type epithelium, smooth muscle fibers, and sometimes of anal glands. This could explain the mucus and fluid discharge. The lack of perianal abscess history in a young patient should prompt this differential diagnostic.

## Data Availability

Not applicable.

## References

[1] Choi S.O., Park W.H. (2003). Anal canal duplication in infants. J. Pediatric Surg..

[2] Koga H., Okazaki T., Kato Y., Lane G.J., Yamataka A. (2010). Anal canal duplication: Experience at a single institution and literature review. Pediatric Surg. Int..

[3] Trecartin A.C., Peña A., Lovell M., Bruny J., Mueller C., Urquidi M., Bischoff A. (2019). Anal duplication: Is surgery indicated? A report of three cases and review of the literature. Pediatric Surg. Int..

[4] Van Biervliet S., Maris E., Vande Velde S., Vande Putte D., Meerschaut V., Herregods N., De Bruyne R., Van Winckel M., Van Renterghem K. (2013). Anal Canal Duplication in an 11-Year-Old-Child. Case Rep. Gastrointest. Med..

[5] Toyonaga T., Matsuda H., Mibu R., Tominaga Y., Hirata K., Takeyoshi M., Tsuneyoshi M. (2018). Anal Canal Duplication Associated with Presacral Cyst in an Adult. J. Anus Rectum Colon.

[6] Ochiai K., Umeda T., Murahashi O., Sugitoh T. (2002). Anal-canal duplication in a 6-year-old child. Pediatric Surg. Int..

[7] Mirzaei R., Mahjubi B., Alvandipoor M., Karami M.Y. (2015). Late presentation of anal canal duplication in adults: A series of four rare cases. Ann. Coloproctol..

[8] Gulen M., Leventoglu S., Ege B., Mentes B.B. (2016). Anal Canal Duplication in Adults: Report of Five Cases. Int. Surg..

[9] Grandjean J.P., Mantion G.A., Guinier D., Henry L., Cherki S., Passebois L., Chalabreysse P., Viennet G., Pujol B., Chabanon J. (2008). Vestigial retrorectal cystic tumors in adults: A review of 30 cases. Gastroenterol. Clin. Et Biol..

[10] Sakr A., Kim H.S., Han Y.D., Cho M.S., Hur H., Min B.S., Lee K.Y., Kim N.Y. (2019). Single-center Experience of 24 Cases of Tailgut Cyst. Ann. Coloproctol..

[11] Lisi G., Illiceto M.T., Rossi C., Broto J.M., Jil-Vernet J.M., Lelli Chiesa P. (2006). Anal canal duplication: A retrospective analysis of 12 cases from two European pediatric surgical departments. Pediatric Surg. Int..

[12] Dukes C.E., Galvin C. (1956). Colloid carcinoma arising within fistulae in the anorectal region. Ann. R. Coll. Surg. Engl..

[13] Tiryaki T., Senel E., Atayurt H. (2006). Anal canal duplicationin children: A new technique. Pediatric Surg. Int..

[14] Levitt M.A., Peña A. (2007). Anorectal malformations. Orphanet J. Rare Dis..

